# Clinorotation-induced autophagy via HDM2-p53-mTOR pathway enhances cell migration in vascular endothelial cells

**DOI:** 10.1038/s41419-017-0185-2

**Published:** 2018-02-02

**Authors:** Cheng-Fei Li, Jia-Xing Sun, Yuan Gao, Fei Shi, Yi-Kai Pan, Yong-Chun Wang, Xi-Qing Sun

**Affiliations:** 1 Department of Aerospace Biodynamics, School of Aerospace Medicine, Fourth Military Medical University, Xi’an, 710032 China; 2 Key lab of Aerospace Medicine, Chinese Ministry of Education, Xi’an, Shaanxi 710032 China

## Abstract

Individuals exposed to long-term spaceflight often experience cardiovascular dysfunctions characterized by orthostatic intolerance, disability on physical exercise, and even frank syncope. Recent studies have showed that the alterations of cardiovascular system are closely related to the functional changes of endothelial cells. We have shown previously that autophagy can be induced by simulated microgravity in human umbilical vein endothelial cells (HUVECs). However, the mechanism of enhanced autophagy induced by simulated microgravity and its role in the regulation of endothelial function still remain unclear. We report here that 48 h clinorotation promoted cell migration in HUVECs by induction of autophagy. Furthermore, clinorotation enhanced autophagy by the mechanism of human murine double minute 2 (HDM2)-dependent degradation of cytoplasmic p53 at 26S proteasome, which results in the suppression of mechanistic target of rapamycin (mTOR), but not via activation of AMPK in HUVECs. These results support the key role of HDM2–p53 in direct downregulation of mTOR, but not through AMPK in microgravity-induced autophagy in HUVECs.

The exposure to microgravity affects almost all systems of human body. Mounting evidence has shown that bone loss^1^, immune dysregulation^2^, cardiovascular deconditioning^3^ and skeletal muscle atrophy occur in spaceflight or simulated microgravity conditions^4^. Among these changes, cardiovascular deconditioning such as post-spaceflight orthostatic intolerance^5^ and atherosclerosis^6^ seriously threatens crewmembers’ health and thus constrains long-duration spaceflight. Endothelial cells which form the inner luminal layer of blood vessels play a pivotal role in vascular functions^7^ through participating in the regulation of smooth muscle contractions, vascular wall permeability, platelet aggregation, inflammatory cells adhesion as well as angiogenesis^8^. It has been demonstrated that cardiovascular deconditioning is closely related to morphology and functional changes of endothelial cells during and after spaceflight^9^ including increase of eNOS and nitric oxide production^10,11^, cytoskeletal lesions, ultrastructural changes, decreased metabolism and altered gene expression^12,13^. However, the underlying mechanism of the functional changes in endothelial cells under microgravity remains to be elucidated yet.

Autophagy, also called cellular self-digestion, is a cellular pathway involved in protein and organelle degradation, which is important for maintaining normal cellular homeostasis by supplying amino acids and energy through catabolism in the cellular response to stress^14^. There are three types of autophagy: chaperone-mediated autophagy, microautophagy, and macroautophagy^15^. The macroautophagy which consists of two consecutive phases is widely studied. The early stage is characterized by the formation of autophagosome namely double-membrane vesicles. The late stage, also known as maturity and degradation stage, mainly involves fusion of autophagosome and lysosome. Comprehensive investigations have demonstrated autophagy occurs in a number of cardiovascular diseases^16^ such as cardiac arrhythmias, ischemia reperfusion injury and diabetic heart. Furthermore, autophagy also involves in the endothelial dysfunction^17,18^. For example, autophagy promotes the cholesterol efflux from macrophage foam cells and prohibits atherosclerotic lipid accumulation^19^. In addition, it is found that autophagy plays a critical role in maintaining NO generation and bioavailability as well as regulating oxidant–antioxidant balance and inflammatory–anti-inflammatory balance when endothelial cells are exposed to shear stress^20,21^. Besides, autophagy disrupts endothelial barrier through degradation of claudin-5 from the cellular cytoskeletal framework after oxygen-glucose deprivation^22^. Moreover, it has been reported that autophagy provides protection against cell death by clearing oxidized low-density lipoprotein in human umbilical endothelial cells^23^ while promotes cell death induced by carbamylated low-density lipoprotein in human coronary endothelial cells^24^. In our previous work, we found that clinorotation simulated microgravity enhances autophagy in vascular endothelial cells^25^. However, the mechanism of enhanced autophagy induced by simulated microgravity and its role in regulating endothelial function still remain unclear.

Vasculogenesis is essential in embryonic development, wound healing, tumor growth, and maintaining the integrity of blood vessel^26^. The migration of endothelial cells is the most early and pivotal event of vasculogenesis^27^. It has been reported that microgravity influence angiogenesis and cell migratory capacities^28–32^. Previous studies have demonstrated that autophagy is closely related to cell migration^33–36^. However, to the best of our knowledge, no prior report has investigated the relationship between autophagy and cell migration under microgravity condition.

The purpose of the present study was to investigate the underlying molecular mechanism of autophagy induced by microgravity and its functional role in the regulation of cell migration. In this study, we show that clinorotation induces HDM2-mediated degradation of p53, which subsequently inactivates mTOR and induces autophagy in HUVEC. We also provide evidence for the functional importance of autophagy in enhancing cell migratory capacity. Our study provides important insights into the functional changes in endothelial cells under microgravity conditions.

## Results

### Clinorotation-induced autophagy in HUVECs

**Fig. 1:**
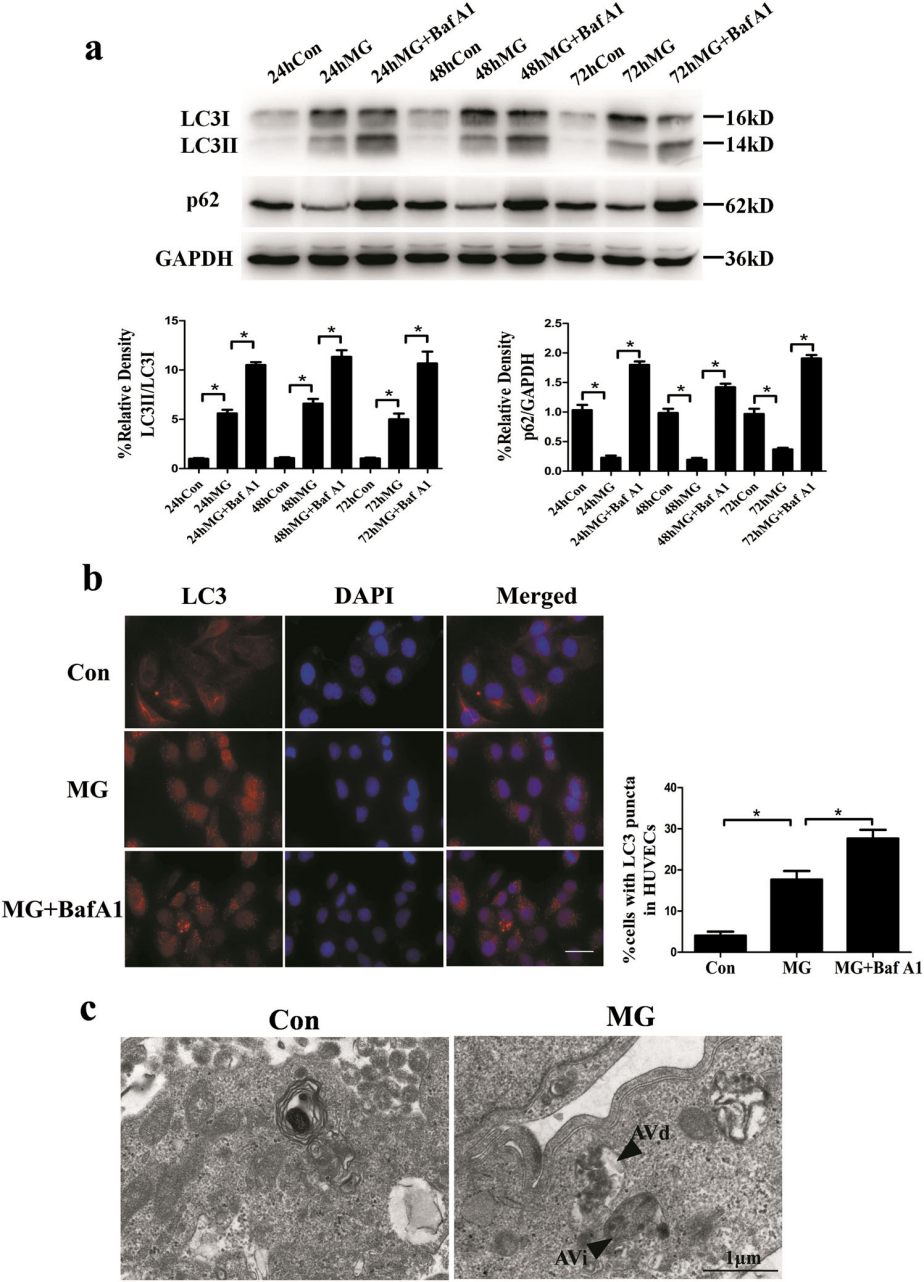
Time-course of the expression of LC3 and p62 in HUVECs shown by western blot analysis and the autophagy induction shown by immunofluorescence and TEM in HUVECs after clinorotation for 48 h. **a** Western blot analysis for the expression of LC3 and p62 in HUVECs after clinorotation for 24, 48, and 72 h with or without Baf A1 (20 nM). **b** Immunostaining of LC3 in HUVECs after clinorotation for 48 h with or without Baf A1 (20 nM) and the percentage of cells containing LC3 puncta (>5). **c** The early initial autophagic vacuoles (AVi) and the degradative autophagic vacuoles (AVd) are shown by arrows in HUVECs after clinorotation for 48 h. The data are expressed as the mean ± s.d. of three replicates each. *p < 0.05 vs. the control. Scale bar: 20 μm

Microtubule-associated protein 1A light chain 3 (LC3) is initially synthesized in an unprocessed form, pro LC3, which is converted into a proteolytically processed form lacking amino acids from the C terminus, LC3I. LC3I is finally modified into the phosphoethanolamine (PE)-conjugated form, LC3II. The ratio of LC3II/LC3I reflects an increase of autophagy. p62 mediates the degradation of protein in autolysosomes and decreased p62 is an indicator of autophagy activation^37^. To investigate whether simulated microgravity enhances autophagy in HUVECs, the expression of LC3 and p62 was examined by western blot after clinorotation for 24, 48, and 72 h. As shown in Fig. 1a, clinorotation for 24, 48, and 72 h increased the ratio of LC3II/LC3I and decreased p62 levels as compared to 1 G control group. Moreover, the ratio of LC3II/LC3I and the amount of p62 were higher compared to those in the microgravity group(MG) in the presence of bafilomycin A1 (BafA1, 20 nM), which disrupts autophagic flux by independently inhibiting acidification and autophagosome–lysosome fusion^38^. Then we chose 48 h as the time point because there were no obvious difference about autophagic level among 24, 48, and 72 h. To further confirm the increased autophagy after clinorotation, we performed immunofluorescence staining to analyze the LC3 puncta. As shown in Fig. 1b, immunofluorescence analysis showed that the number of cells which contained more than 5 puncta of LC3 increased after clinorotation for 48 h. In addition, 48 h clinorotation with treatment of Baf A1 (20 nM) resulted in a marked increase in the number of cells containing more than 5 puncta of LC3 compared to MG group, suggesting the occurrence of autophagic flux under clinorotation. The enhanced autophagic level was also revealed by transmission electron microscope (TEM). As shown in Fig. 1c, autophagosomes and autolysosomes were found in HUVECs after clinorotation for 48 h. Taken together, these results suggested that clinorotation induces autophagy in vascular endothelial cells.

### The autophagy induced by microgravity enhanced cell migration of HUVECs

**Fig. 2:**
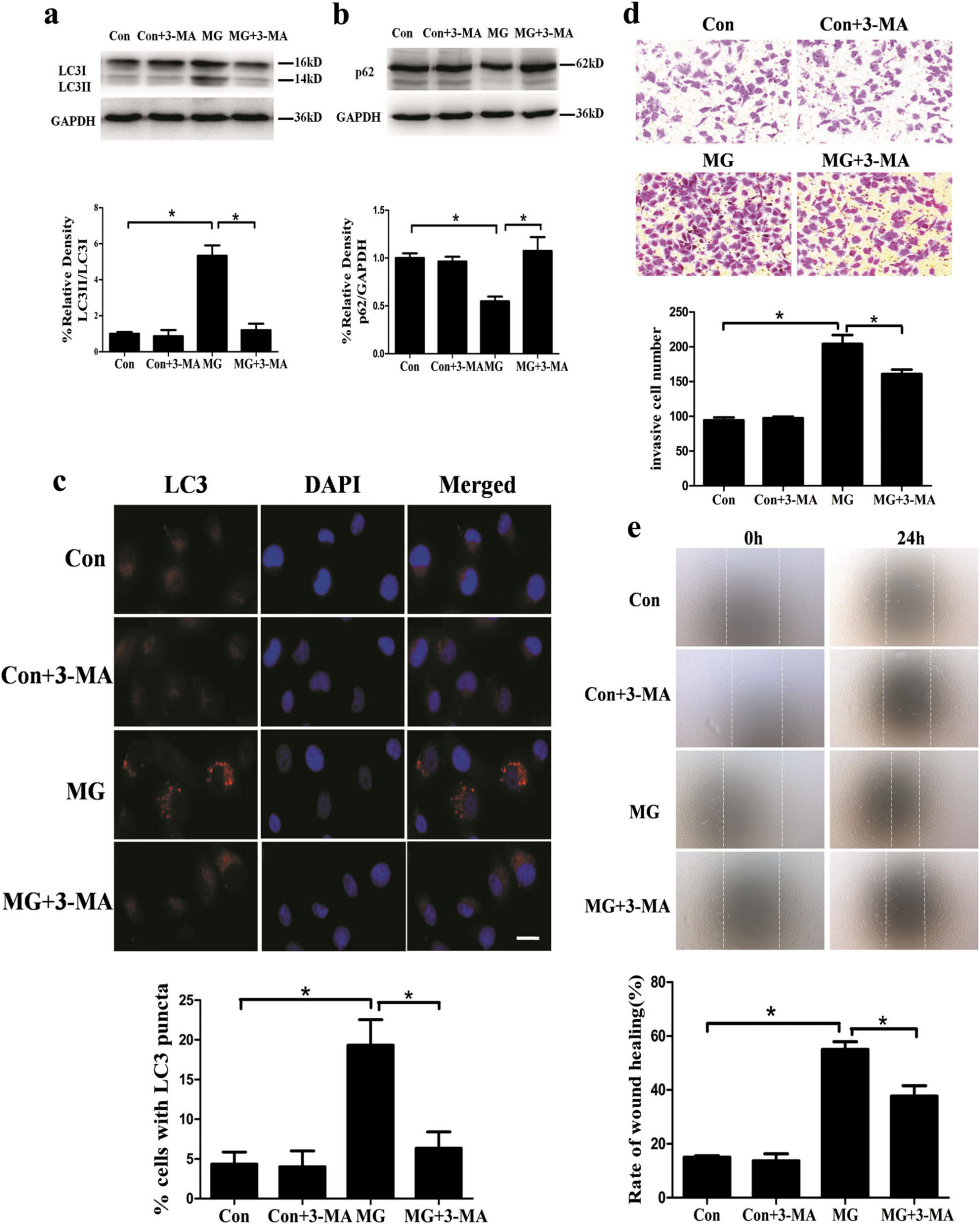
Autophagy enhanced cell migration after simulated microgravity. **a**, **b** Western blot analysis for expressions of LC3 and p62 in HUVECs with or without 3-MA (10 mM) after clinorotation for 48 h. **c** Immunostaining of LC3 in HUVECs after clinorotation for 48 h with or without 3-MA and the percentage of cells containing LC3 puncta (>5).** d** Transwell migration assay. Cells were treated with or without 3-MA while exposed to microgravity for 48 h, and then Transwell migration assay was performed and the migrated cells were fixed and stained. Representative photos of migrated HUVECs were observed using the microscope.** e** Wound-healing assay. Cells were exposed to simulated microgravity for 48 h with or without 3-MA. Then the scratches were made and cultured for 24 h. Microphotographs of the scratches were obtained as soon as the scratches were made and after 24 h. The data are expressed as the mean ± s.d. of three replicates each. *p < 0.05 vs. the control. Scale bar: 20 μm

Directional migration of endothelial cell, essential for angiogenesis and structural integrity of vascular walls, is regulated by chemotactic, haptotactic, and mechanotactic stimuli^10^. To evaluate the functional role of autophagy in endothelial cell migration, the autophagy inhibitor 3-methyladenine (3-MA, 5 mmol/L) was used to inhibit the autophagy under the clinorotation condition. 3-MA has been widely used as autophagy inhibitors due to the inhibitory effect on class III PI3K, which stimulates autophagy. However, it must be kept in mind that this compound also has stimulatory effects on autophagy under some conditions^39^. Therefore, our initial intention was to investigate the effect of 3-MA on autophagy induced by clinorotation in HUVECs. As shown in Fig. 2a, b, 3-MA prevented the effects of clinorotation on the increase in the ratio of LC3II/LC3I and the decrease in p62 levels in HUVECs as confirmed by western blot. Consistent with the Western blot results, immunofluorescence displayed decreased level of the autophagy, which was revealed by the decreased number of cells containing >5 puncta of LC3 in HUVECs after clinorotation for 48 h with 3-MA as compared to the MG group (Fig. 2c). These results showed that 3-MA can effectively inhibit clinorotation-induced autophagy in HUVECs.

To determine the role of autophagy in regulating cell migration in HUVECs under clinorotation, the Transwell assay and wound-healing assay were performed. As shown in Fig. 2d, the number of the migrated cells in MG group was significantly higher than that of the control group and the presence of 3-MA reduced the number of migrated cells. Consistently, the rate of wound healing was higher in MG group than the control group and the treatment of HUVECs with 3-MA under clinorotation reduced the percentage of wound healing compared to the MG group (Fig. 2e). These results suggested that autophagy in HUVECs induced by clinorotation enhances the cell migration.

### p53-mTOR signaling pathway was involved in the clinorotation-induced autophagy in HUVECs

**Fig. 3:**
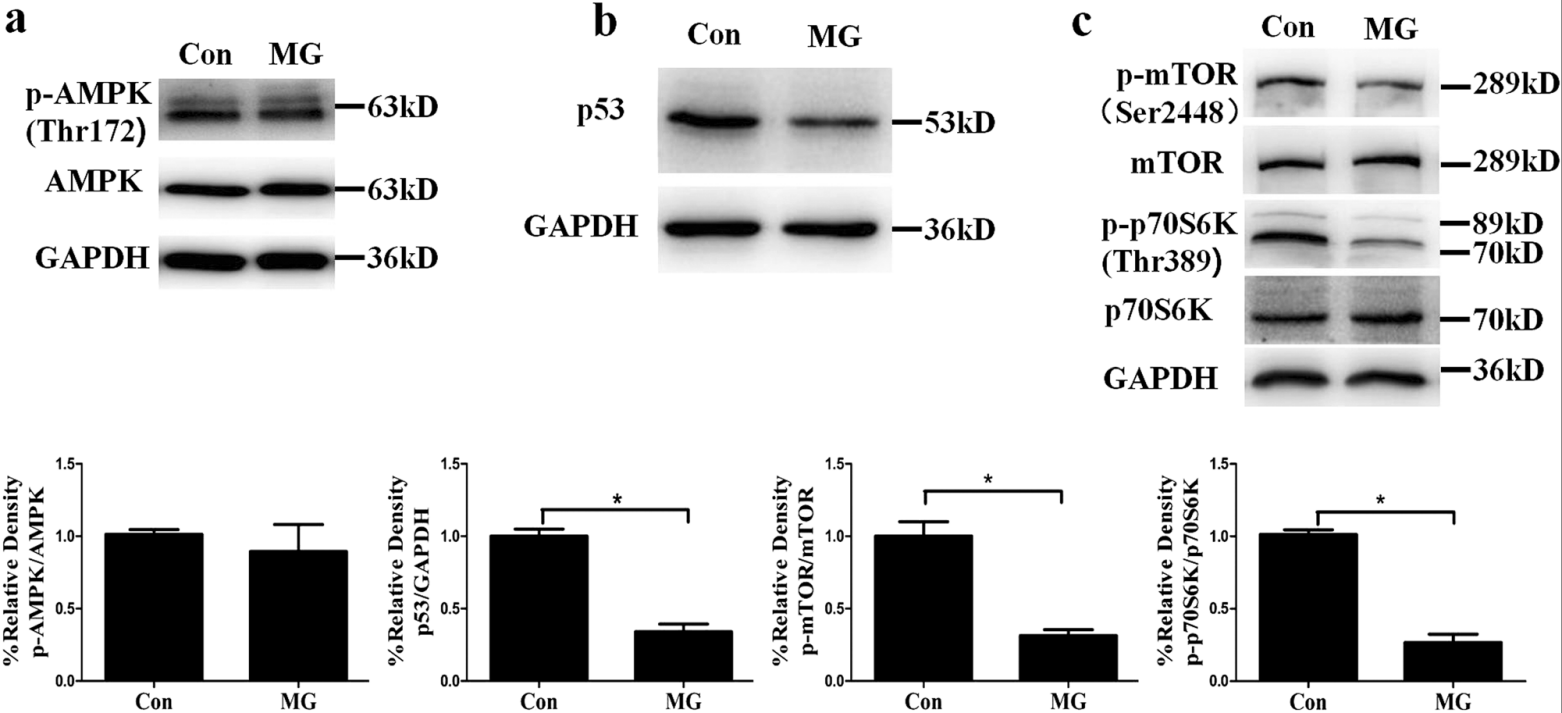
The expression of p-AMPK, p53, p-mTOR and p-p70S6K in HUVECs after exposure to clinorotation or stationary condition for 48 h. **a**,** b**, **c** Western blot analysis for protein levels of p-AMPK (**a**), p53 (**b**), p-mTOR and p-p70S6K (**c**) after clinorotation simulated microgravity for 48 h.The data are expressed as the mean ± s.d. of three replicates each. *p < 0.05 vs. the control

**Fig. 4:**
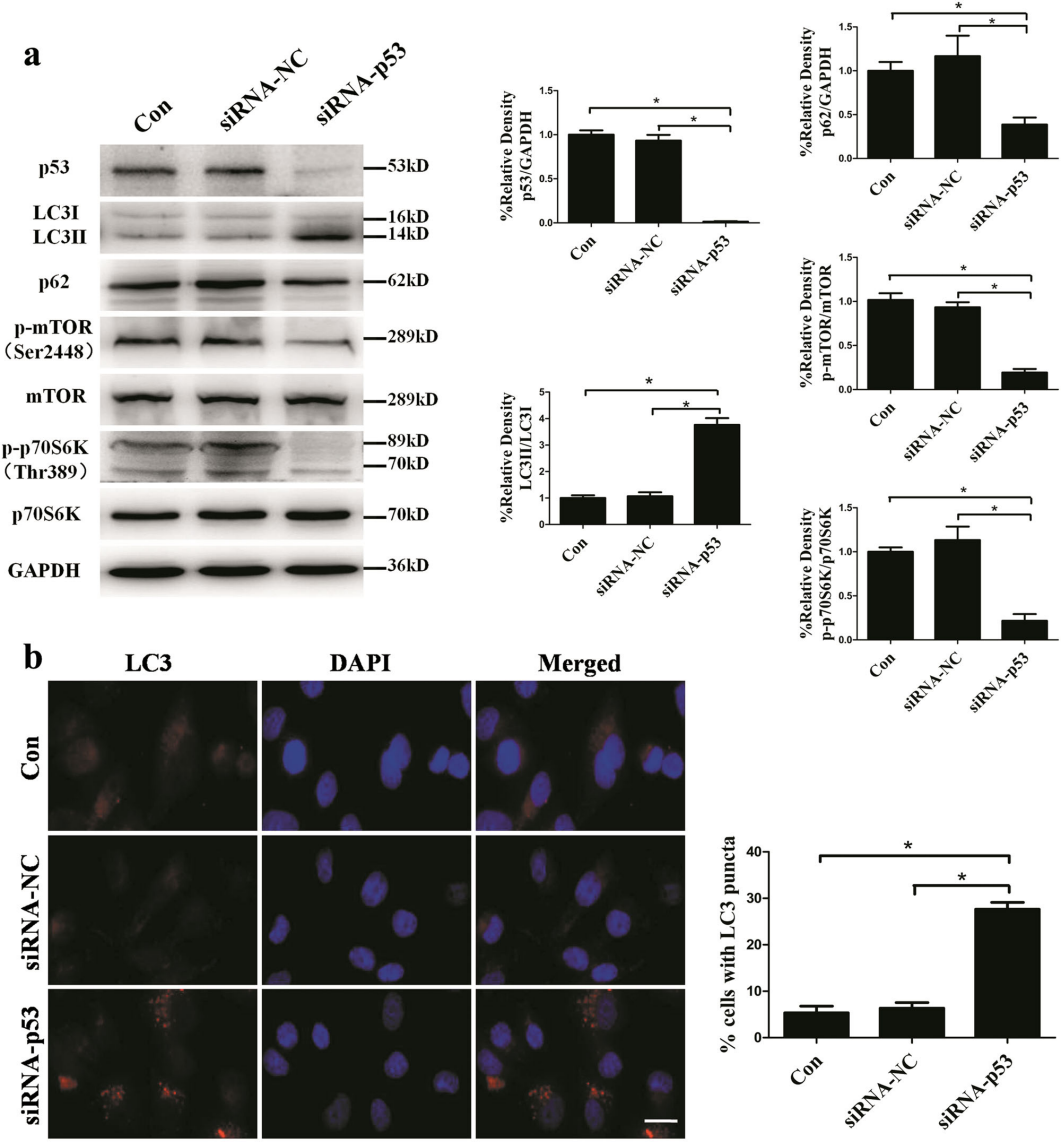
The autophagic changes in HUVECs after p53 knockdown under stationary condition. **a** HUVECs were transfected with FAM-siRNA. The cells in bright field and the corresponding dark field were examined under the fluorescence microscope. **b** Western blot analysis for expressions of LC3, p62, p53, p-mTOR, and p-p70S6K after transfection with siRNA-p53 or siRNA-NC under stationary condition for 48 h. **c** Immunostaining of LC3 in HUVECs after transfection with siRNA-p53 or siRNA-NC under stationary condition for 48 h and the percentage of cells containing LC3 puncta (>5). The data are expressed as the mean ± s.d. of three replicates each. *p < 0.05 vs. the control. Scale bar: 20 μm

**Fig. 5:**
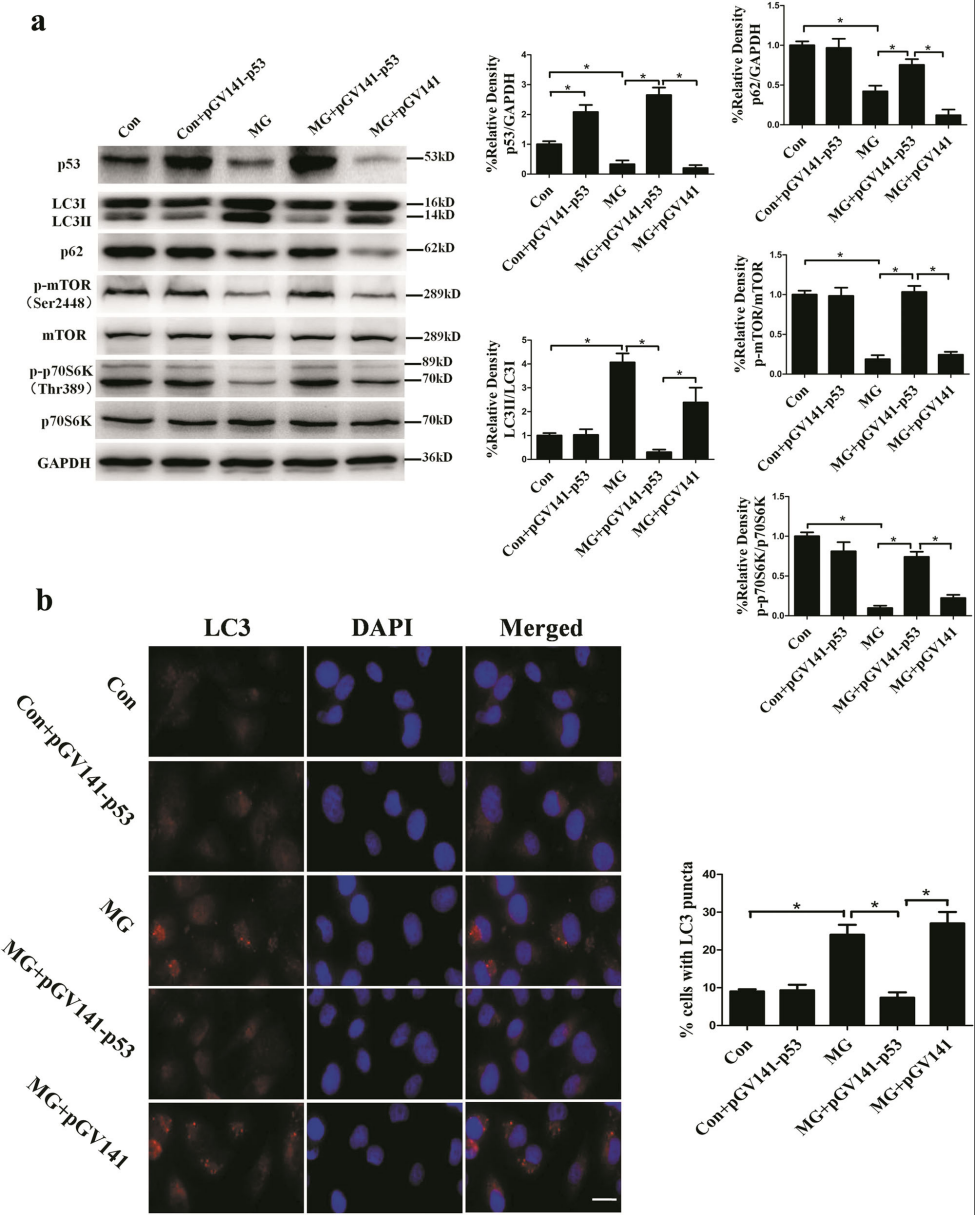
The autophagic changes in HUVECs after overexpression of p53 under simulated microgravity condition. **a** Western blot analysis of the changes in protein expression of LC3, p62, p53, p-mTOR, and p-p70S6K after transfection with pGV141-p53 or pGV141 under simulated microgravity for 48 h. **b** Immunostaining of LC3 in HUVEC after transfection with pGV141-p53 or pGV141 under simulated microgravity for 48 h and the percentage of cells containing LC3 puncta (>5). The data are expressed as the mean ± s.d. of three replicates each. *p < 0.05 vs. the control. Scale bar: 20 μm

p53, a tumor suppressor protein, is known to play a dual role in regulating autophagy. On one hand, p53 can be activated and induce autophagy under starvation or genotoxic stress^40,41^. On the other hand, inactivation of p53 by deletion, depletion or inhibition by pifithrin-α can trigger autophagy^42^. AMPK and mTOR as two fuel sensors are two central checkpoints regulating autophagy^43^. The level of the phosphorylation of Thr-172 is indicative of AMPK activity in cells. mTOR specifically leads to the activation of ribosomal protein S6 kinase (p70S6K) and eIF4E-binding protein 1 (4E-BP1). Western blot analysis to determine the phosphorylation of mTOR at Ser-2448 and the phosphorylation of p70S6K at Thr-389 is an established and specific assay used to monitor mTOR activity^44^. Analysis of western blot revealed that the expression of p53, the phosphorylation of mTOR and the phosphorylation of p70S6K were decreased as compared to the control group in HUVECs after clinorotation for 48 h, while no obvious change was observed in the levels of phosphorylation of AMPK (Fig. 3a–c). To further test the role of p53 and mTOR in autophagy induction under clinorotation, the impacts of p53 knockdown and overexpression on the activity of mTOR as well as the levels of autophagy were studied in HUVECs. As shown in Fig. 4a, significantly increased ratio of LC3II/LC3I, decreased level of p62 and phosphorylation of mTOR were observed following the transfection of siRNA-p53 under 1G condition. The percentage of cells containing more than 5 puncta of LC3 was higher than control group as measured by immunofluorescence method(Fig. 4b). Moreover, we observed the level of autophagy in HUVECs with overexpression of p53 during clinorotation for 48 h. The autophagy level in HUVECs was compromised by the p53 overexpression after simulated microgravity for 48 h (Fig. 5). Taken together, these results indicated that clinorotation decreases the expression of p53 in HUVECs, thus inactivating mTOR and inducing autophagy independently of activation of AMPK.

### Clinorotation-induced HDM2-mediated 26S proteasome-dependent degradation of p53 in HUVECs

**Fig. 6:**
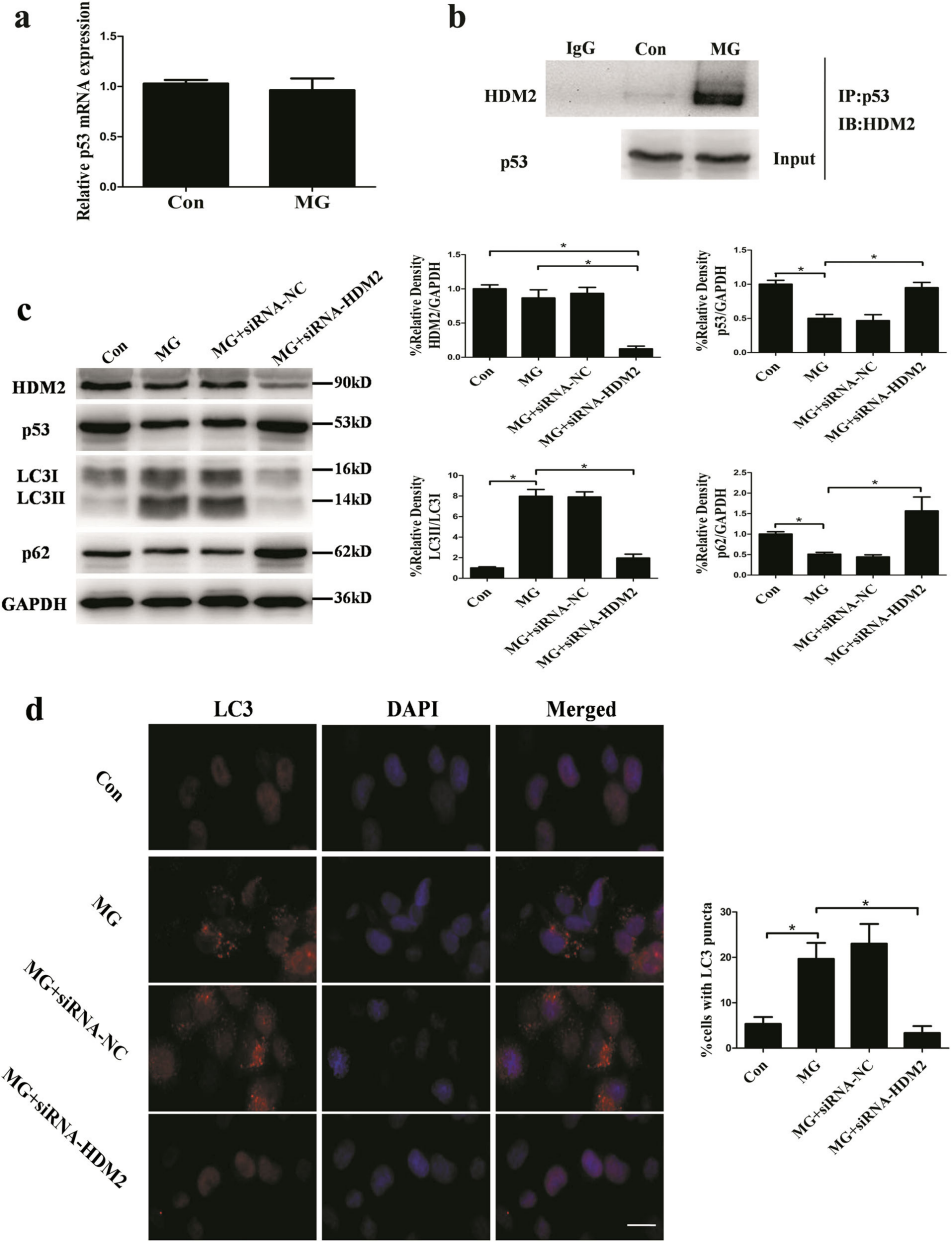
The mRNA expression of p53 after clinorotation in HUVECs and the interaction between p53 and HDM2 after simulated microgravity for 48 h. **a** The mRNA expression of p53 in HUVECs after exposure to clinorotation for 48 h. **b** Co-IP assay for the interaction between p53 and HDM2 after simulated microgravity for 48 h with MG132 (5 μmol/L). **c** Western blot analysis for expressions of LC3, p62, p53, and HDM2 in HUVECs after transfection with siRNA-HDM2 or siRNA-NC under clinorotation for 48 h. **d** Immunostaining of LC3 in HUVECs after transfection with siRNA-HDM2 or siRNA-NC under simulated microgravity for 48 h and the percentage of cells containing LC3 puncta (>5). The data are expressed as the mean ± s.d. of three replicates each. *p < 0.05 vs. the control. Scale bar: 20 μm

**Fig. 7:**
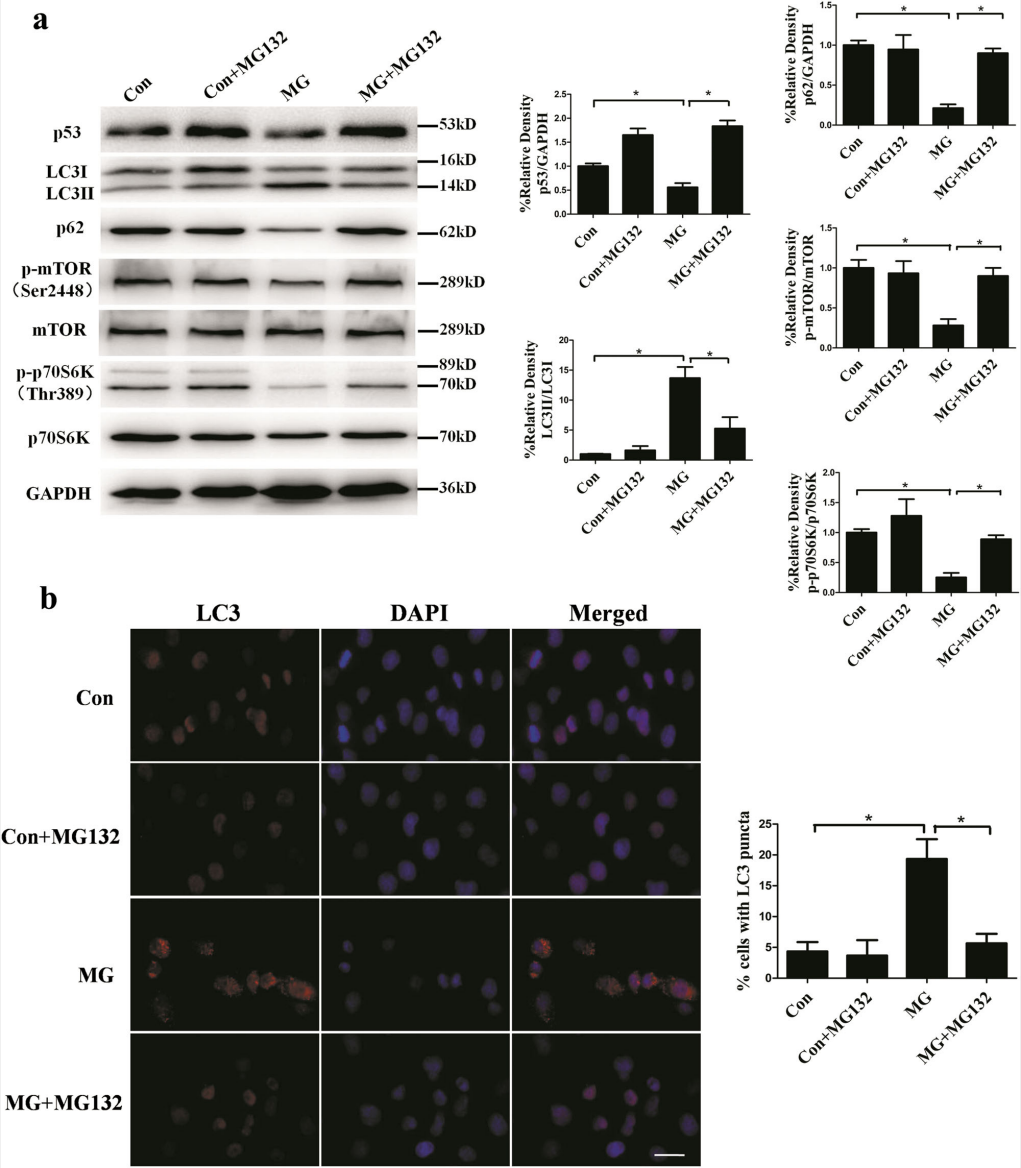
The effects of MG132 on the autophagy induction in HUVECs after clinorotation for 48 h. **a** Western blot analysis for expressions of LC3, p62, p53, p-mTOR, and p-p70S6K in HUVECs with or without MG132 (5 μmol/L) under clinorotation for 48 h. **b** Immunostaining of LC3 in HUVECs after clinorotation for 48 h with or without MG132 (5 μmol/L) and the percentage of cells containing LC3 puncta (>5). The data are expressed as the mean ± s.d. of three replicates each. *p < 0.05 vs. the control. Scale bar: 20 μm

To investigate the mechanism of decreased level of p53 induced by clinorotation, we firstly examined the change of mRNA level of p53 in HUVECs after clinorotation for 48 h. The quantitative real-time PCR (qRT-PCR) analysis showed that there was no significant difference in mRNA level of p53 between MG group and control group (Fig. 6a), whereas clinorotation decreased the protein level of p53 in HUVECs, suggesting an effect of clinorotation on post-transcriptional modifications of p53. Given that the cellular levels of p53 are mainly regulated by HDM2 which can promote ubiquitination and proteasome-dependent degradation of p53 due to its ubiquitin E3 ligase activity^45–47^, the role of HDM2 in the reduction of p53 in HUVECs after clinorotation was determined. To test the requirement of HDM2 for p53 degradation in response to simulated microgravity, we examined the interaction between p53 and HDM2 in HUVECs exposed to clinorotation for 48 h by immunoprecipitation. Cells were treated with 26S proteasome inhibitor MG132 (5 μM) to stabilize p53 in both control and MG group. As seen in Fig. 6b, the association of HDM2 to p53 after clinorotation was stronger than that of HDM2 in control group. Then, we used the siRNA approach to knock down the expression of HDM2. As shown in Fig. 6c, d, transfection with HDM2 siRNA delayed the decreased level of p53 and increased level of autophagy induced by clinorotation in HUVECs as confirmed by western blot and immunostaining of LC3. Furthermore, clinorotation-induced activation of autophagy was blocked by the presence of 26S proteasome inhibitor MG132 (Fig. 7). Collectively, these data strongly indicated that HDM2 mediates proteasome-dependent degradation of p53 in HUVECs under clinorotation condition.

### Cytoplasmic p53 was required for inhibition of autophagy in HUVECs

**Fig. 8:**
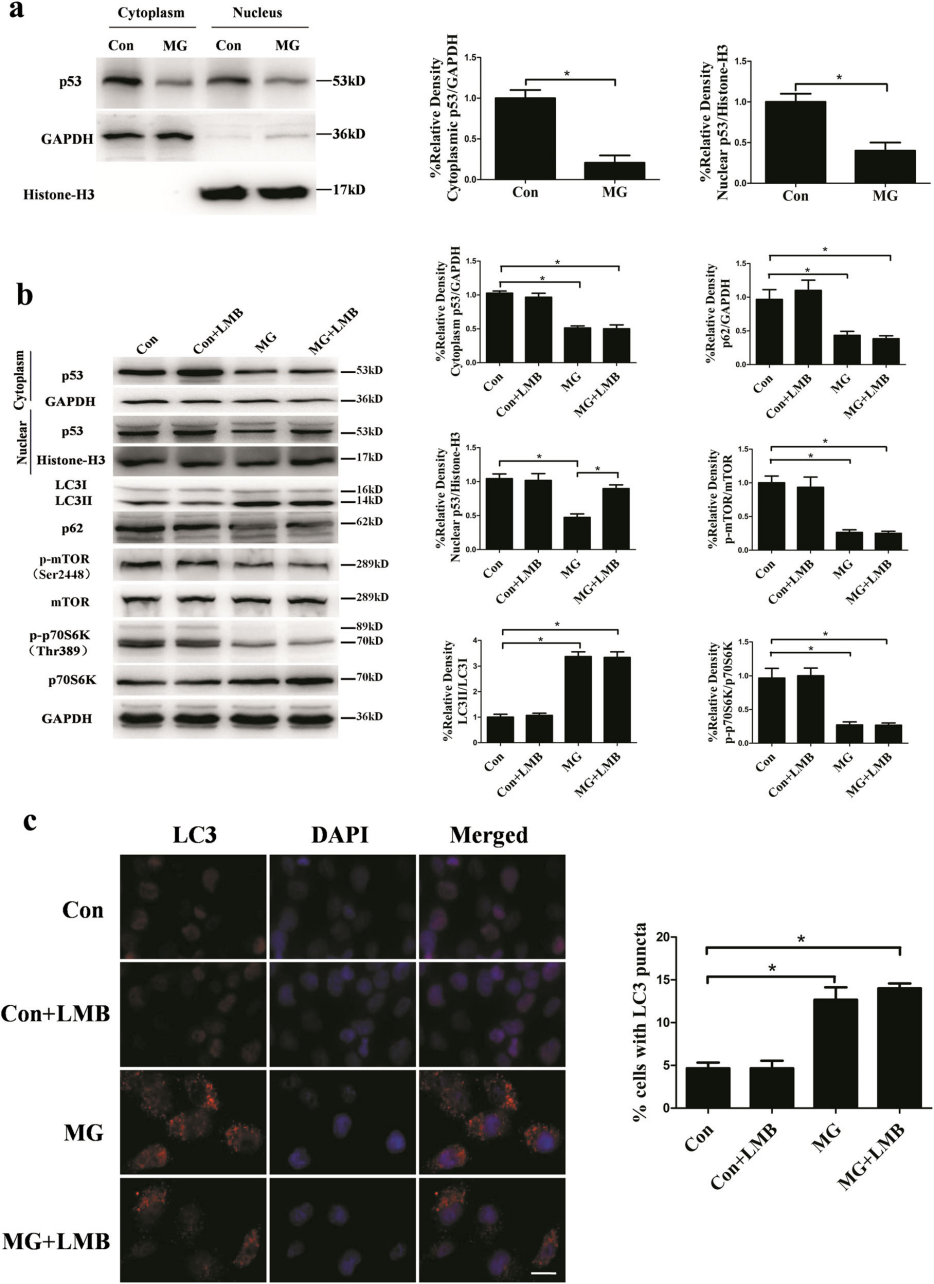
The effects of LMB on the autophagy induction after clinorotation for 48 h in HUVECs. **a** Western blot analysis for expressions of p53 in nucleus and cytoplasm in HUVECs under clinorotation for 48 h. **b** Western blot analysis for expressions of LC3, p62, p-mTOR, p-p70S6K, and p53 in nucleus and cytoplasm in HUVECs with or without LMB (20 μg/L) under clinorotation for 48 h. **c** Immunostaining of LC3 in HUVECs after clinorotation for 48 h with or without LMB (20 μg/L) and the percentage of cells containing LC3 puncta (>5). The data are expressed as the mean ± s.d. of three replicates each. *p < 0.05 vs. the control. Scale bar: 20 μm

It is well established that p53 plays a dual role in the regulation of autophagy and the bidirectional control of autophagy by p53 is closely related to its subcellular localization. p53 in the nucleus can transactivate multiple genes involved in autophagy such as *AMPK, DAPK-1*, and *DRAM*^48^. Inhibition of autophagy by cytoplasmic p53 was accompanied by reduced phosphorylation of AMPK and increased phosphorylation of p70S6K, suggesting the impact of cytoplasmic p53 on the AMPK/mTOR pathway^49^. The data presented above showed reduced expression of p53 and enhanced autophagy under clinorotation, indicating that p53 inhibits autophagy in HUVECs. To understand the effects of p53 in nucleus and cytoplasm on autophagy in HUVECs under simulated microgravity, we first accessed the protein levels of p53 in nucleus and cytoplasm respectively after clinorotation for 48 h in HUVECs. As shown in Fig. 8, clinorotation significantly decreased the levels of p53 in both nucleus and cytoplasm. Moreover, to block nuclear export and stabilize nuclear p53, we treated HUVECs with leptomycin B (LMB, 50 nM) under clinorotation. Despite the protein level of p53 in nucleus remained unchanged in LMB-treated HUVECs compared to control group, the autophagy level was increased after clinorotation for 48 h (Fig. 8). The above data indicated that clinorotation-induced autophagy is caused by the degradation of cytoplasmic p53 in HUVECs.

## Discussion

**Fig. 9:**
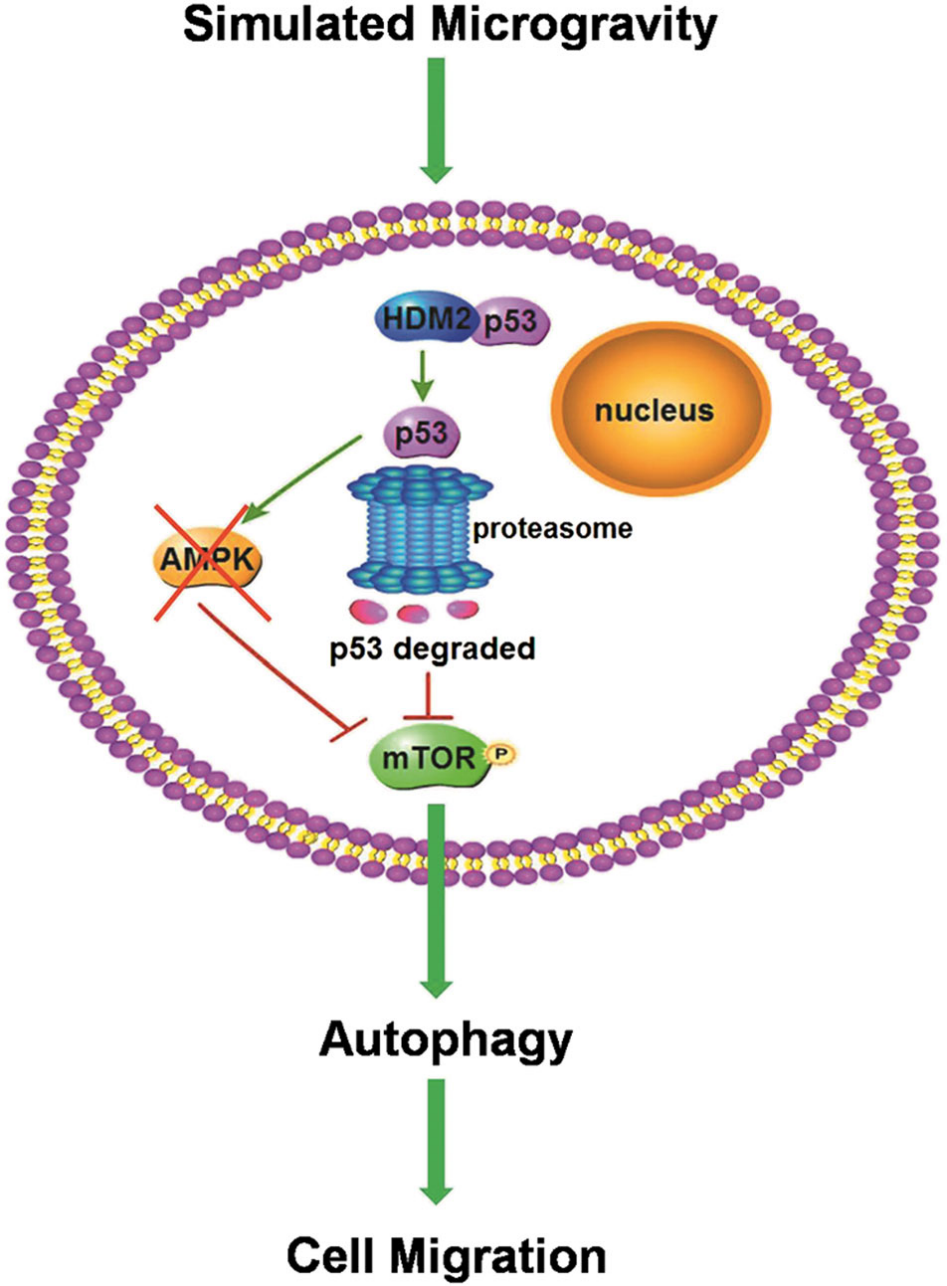
Schematic diagram of autophagy induction by simulated microgravity in HUVECs. When HUVECs are exposed to clinorotation, p53 in cytoplasm and nucleus is degraded at proteasome mediated by HDM2. The degradation of cytoplasmic p53 but not nuclear p53 inactivates mTOR, leading to the induction of autophagy. The autophagy induced by clinorotation enhances the migratory ability of HUVECs

Among the observations demonstrated in this manuscript, the most significant and novel finding in endothelial cells cultured on clinostat, which simulates microgravity was that induction of autophagy promotes cell migration. In addition, for the first time, we found that simulated microgravity induces autophagy via degradation of cytoplasmic p53 mediated by HDM2 which subsequently inactivated mTOR independent of AMPK in human endothelial cells as shown in Fig. 9.

Vascular endothelial cells play a crucial role in the pathogenesis of many diseases and are highly sensitive to microgravity. It is widely known that microgravity causes cytoskeleton disruption, actin fibers redistribution, altered adhesion molecules, changes of apoptosis and proliferation rate, and many other functional changes in endothelial cells^50,51^. Autophagy is one of the two intracellular protein degradation systems (the other is ubiquitin–proteasome system) and can be activated under various stimuli such as starvation and hypoxia. However, whether microgravity as a special stimulus cause autophagy in vascular endothelial cells is largely unknown, although the increased level has been reported^52,53^. Our previous study found that clinorotation for 48 h increases the level of autophagy in HUVECs^25^. The results in this study indicated that the autophagy levels were increased in HUVECs after clinorotation for 24, 48, and 72 h.

The migratory capacity of endothelial cells is essential for vasculogenesis and the structural integrity of vascular walls. It is measured by the wound-healing assay and Transwell assay. It has been reported that low gravitational forces alter the migratory capacity in endothelial cells. Accumulating evidence has shown an increase in cell migration under microgravity^10,31^, even if some reports show opposite results^54^. PI3K-independent NO production, the activation of eNOS and cytoskeletal changes may be the underlying mechanisms, which alter migration in endothelial cells under simulated microgravity^31,55^. In addition, previous studies have reported the role of autophagy in cell migration and invasion ability but there are conflicting data on the involvement of autophagy in the regulation of cell migration. HIF-1α and TGF-β1 promotes cell invasion and migration by inducing autophagy^33,34^ and let-7 activates radial migration of new-born neurons by enhancing autophagy^35^, whereas metformin enhances the autophagy levels and thus inhibits the migration of endothelial progenitor cells^36^ and autophagy induction impairs migration and invasion in glioblastoma cells^56^. Our results demonstrate that clinorotation enhances cell migration through inducing autophagy in HUVECs. Astronauts under microgravity may experience injury and the results obtained here will help determine a potential treatment to accelerate new blood vessel development, which is a key factor of wound and tissue repair. However, according to our results, the other underlying mechanism of the change of cell migration apart from autophagy can not be excluded and remains to be further elucidated in the future.

The regulation of autophagy is incredibly complex. The molecules and pathways involved in autophagy are different under different environmental stimuli. p53 as a tumor suppressor protein can maintain the genetic stability by repairing DNA, arresting cell cycle, inducing senescence and apoptosis^57^. p53 plays a critical but ambiguous role in the regulation of autophagy. On one hand, several preautophagic factors such as DRAM, DAPK-1, and PUMA are transactivated by p53 and autophagy is induced by upregulating p53 under genotoxic stress or other conditions as is evidenced by the fact that inhibition of p53 by pifithrin-α or siRNA of p53 attenuates the autophagy induction^48,58^. On the other hand, Tasdemir *et al*. found that inactivation of p53 by gene knockout, RNA interference or chemical agents induced autophagy^49^. Our data demonstrate that p53 negatively regulates autophagy and decreased protein level of p53 leads to autophagy under clinorotation condition in HUVECs.

Many signaling ways regulating autophagy converge on the positive regulator AMPK, an conserved sensor of cellular energy levels and negative regulator mTOR, which regulates cell growth. AMPK promotes autophagy by at least the following ways: phosphorylating the tuberous sclerosis (TSC) complex proteins TSC1 and TSC2, which in turn downregulate mTOR activity and induce autophagy^59^, phosphorylating FOXO3, phosphorylating ULK1, and dissociating Beclin1 and Bcl-2 by stimulating JNK1-Bcl-2 signaling^60–62^. mTOR is controlled by growth factors via Akt/PKB and cell energy status via AMPK. However, it is worth noting that inhibition of mTOR by amino-acid starvation or rapamycin treatment induces autophagy in an AMPK-independent manner^63^. In the present study, we observed significant reduction in the activity of mTOR but no obvious changes in the phosphorylation of AMPK as a result of exposure to simulated microgravity in HUVECs. These results indicate that the decreased expression of p53 in HUVECs after clinorotation for 48 h induces autophagy through decreasing the activity of mTOR independent of AMPK.

It is interesting to investigate the reason why clinorotation causes decreased expression of p53. The finding that there are no obvious changes at the mRNA level suggests post-transcriptional regulation of p53 under clinorotation in HUVECs. It has been known that HDM2, one of the E3 ubiquitin ligases, is a major regulator of p53 stability inducing p53 polyubiquitination and degradation at 26S proteasome^64^. Previous study shows that various inducers of autophagy such as ER stress, rapamycin and nutrient starvation cause HDM2-dependent p53 degradation^49^. However, it is not presently known whether HDM2 is the determinant of p53 degradation under clinorotation in HUVECs. In our study, the decreased activity of mTOR and the enhanced autophagy in HUVECs induced by clinorotation were inhibited by the HDM2 knockout and the 26S proteasome inhibitor MG132. Moreover, the intermolecular interaction of the p53 and HDM2 is enhanced under microgravity conditions. These data provide strong evidence that p53 is degraded at 26S proteasome after binding to HDM2 during clinorotation for 48 h, thus decreasing the activity of mTOR and inducing autophagy in HUVECs. In this study, we also demonstrate the reduction of both nuclear and cytoplasmic p53 after clinorotation, which supports the notion that nuclear export is not required for p53 degradation by HDM2^65^. In addition, given that the dual role of p53 in autophagy is related to the subcellular localization and nuclear p53 stimulates the autophagic pathway, whereas cytoplasmic p53 inhibits autophagy^49^, it is important to determine the role of reduced nuclear and cytoplasmic p53 in the regulation of autophagy under clinorotation. In our study, we used LMB to inhibit HDM2-mediated degradation of nuclear p53 but not cytoplasmic p53. The autophagy induced by simulated microgravity was not reversed in HUVECs treated with LMB, demonstrating that the degradation of cytoplasmic p53 is required for induction of autophagy. Although the HDM2-dependent p53 degradation is critical for autophagy induction, the molecular mechanisms initiating this process merit further exploration. Future investigation is to determine whether ER-stress leads to HDM2-dependent p53 degradation under clinorotation because HDM2-dependent degradation of p53 is enhanced in ER stressed cells^66^ and ER-stress is found under simulated weightlessness^53,67^.

In summary, the experiments performed in this study established that simulated microgravity enhances cell migration by activating endothelial autophagy in macrovascular endothelial cells. The work also demonstrated that the proteasome-dependent degradation of cytoplasmic p53 mediated by HDM2 is the leading cause of autophagy in the effects of microgravity on macrovascular endothelial cells. Finally, the dissection of autophagy upstream signaling brought to light the key role of p53-mTOR pathway in microgravity-induced autophagy. This study supports the role of autophagy in functional changes in endothelial cells under clinorotation and offers new insights into the mechanisms of cardiovascular dysfunction under microgravity.

## Materials and methods

### Cell culture and drug treatments

HUVECs were purchased from ATCC and cultured in RPMI1640 medium, supplemented with 10% fetal bovine serum (Invitrogen, Carlsbad, CA, USA) at 37 °C in humidified air containing 5% CO_2_. The autophagy inhibitor 3-methyladenine (3-MA, MP, CA, USA) was dissolved in ddH_2_O at concentration of 200 mM for storation at room temperature and bafilomycin A1 (Baf A1, Abcam, Cambridge, UK) was dissolved in dimethyl sulfoxide at concentration of 400 mM and kept in 4 °C. The proteasome inhibitor MG132 (Targetmol, MA, USA) was dissolved in dimethyl sulfoxide at stock concentration of 5 mM and kept in −20 °C.The nucleocytoplasmic transport inhibitor leptomycin B (LMB, Beyotime, Shanghai, China) was prepared at concentration of 0.2 mg/mL and stored at −20 °C.The final concentrations of the drugs and the duration of treatments are indicated in the figure legends.

### Simulated microgravity

Clinorotation is recognized as an effective method of simulated microgravity on the ground. The 2D-clinostat used in these experiments models certain aspects of microgravity, and similar changes in the structure of the cytoskeleton have been observed under clinorotation as in flight^68^. Our previous results documenting functional alterations in HUVECs cultured in the clinostat mimic the results obtained in true microgravity, suggesting the validity of using this surrogate ground-based system for microgravity research^11,25,31,69^. In short, cells were seeded at a density of 1 × 10^5^ cells per well in six-well plates each of which contained a coverslip 2.55 × 2.15 cm in size. Then the cells were cultured at 37 °C in a humidified incubator with 5% CO_2_ before they adhered to coverslips. The coverslips were placed into the fixture of the chambers (Astronaut Research and Training Center, Beijing, China), which were subsequently filled completely with culture medium. Air bubbles were removed to eliminate the effects of shear stress. Then, the chambers rotated around the horizontal axis at 30 rotations/min for 24, 48, and 72 h with or without Baf A1. The continuous rotation changes the direction of gravity applied to the coverslips and the gravity can not be sensed by cells, thus creating the effects on cells similar to actual microgravity. The gravitational force acting on the HUVECs is about 10^−3^ when the clinostat rotates at 30 rotations/min. The cells exposed to clinorotation were used as microgravity group (MG) and the paralleled stationary chambers were used as control group to eliminate effects of other factors. The cells under clinorotation in the presence of Baf A1 were used as MG + Baf A1 group. The clinostat and the stationary chambers were put in an incubator at 37 °C.

### In vitro plasmid and siRNA transfection

HUVECs at 80% confluence were transfected in six-well plates with siRNA targeting p53(NM_000017.11) and HDM2 (NM_000012.12) or siRNA-NC by Lipofectamine 2000 (Invitrogen). HUVECs were also transfected with pGV141-p53 or pGV141.Cells were lysed for protein expression by western blot or subjected to immunofluoresence 48 h after transfection with p53 siRNA. HUVECs were subjected to 48 h clinorotation after transfection with HDM2 siRNA and pGV141-p53 prior to protein expression analysis and immunofluorescence.

### Transmission electron microscopy analysis

Cells were fixed for 30 min with 2.5% glutaraldehyde in 0.1 M cacodylate buffer, embedded in Epoxy resin, and processed for transmission electron microscopy by standard procedures.

### qRT-PCR

Total RNA was prepared using TRIzol kit (Invitrogen) according to the manufacturer’s instructions and 1 μg of total RNA was reverse-transcribed to cDNA using the PrimeScriptRT reagent Kit (Takara, Tokyo, Japan). qRT-PCR was performed using a CFX96 (Bio-Rad, Hercules, CA, USA) instrument and SYBR Premix Ex Taq (Takara) following the manufacturer’s instructions. The data were analyzed via the relative Ct method and were expressed as a fold change compared with the control. Each qRT-PCR was performed by using four biological replicates in triplicate each. The following primers were used:

5′-GCTCGACGCTAGGATCTGAC-3′/5′-CAGGTAGCTGCTGGGCTC-3′(human p53) and 5′-AAAGGTGGAGGAGTGGGT-3′/5′-GGGAAACTGTGGCGTGAT-3′ (human GAPDH). All values obtained were normalized to the values obtained for GAPDH.

### Transwell migration assay

Cell migration was assessed using a two-chamber Transwell system (Corning, NY, USA). HUVECs (2 × 10^5^ cells/mL) in serum-free medium were planted on insets and 600 μL of the RPMI 1640 medium with 10% fetal bovine serum were added to the lower chambers. After incubation at 37 °C in 5% CO_2_ for 24 h, cells that did not migrate through the filter were removed with a cotton swab. Migrating cells that were attached to the bottom of the membrane were fixed with 4% paraformaldehyde for 15 min and stained with crystal violet for 20 min. The cells on the membrane were observed using an inverted microscope (Olympus, Tokyo, Japan) at ×200 magnification. The numbers of the migrating cells in each well were counted in five random microscopic fields per membrane. The experiments were performed in triplicate independently.

### Wound-healing assay

After clinorotation for 48 h, scratches were made on the coverslips using a sterile 200 μL pipette tip and cell debris was washed away with PBS. The HUVECs on coverslips were cultured in serum-free RPMI 1640 medium in a humidified 5% CO2 incubator at 37 °C for 24 h. Wound width was measured under inverted microscope at time zero and 24 h later respectively.

### Cellular fractionation

Nuclear and cytoplasmic fractions were extracted by using the Nuclear and Cytoplasmic Protein Extraction Kit (Beyotime) according to the manufacturer’s instructions.

### Immunoprecipitation and western blot

For immunoprecipitation, cell lysates were incubated with the anti-p53 antibody (1:1000, Proteintech, Illinois, USA) and protein A-agarose beads overnight at 4 °C. Beads were washed and bound proteins were eluted in loading buffer and then analyzed by western blot using anti human murine double minute 2 (HDM2) (1:1000, Abcam, Cambridge, UK) antibody. Western blot were performed as follows. Cells were washed with ice-cold PBS and scraped in the presence of radioimmunoprecipitation (RIPA) buffer containing 1 mM phenylmethylsulfonyl fluoride (PMSF, Beyotime) and phosphatase inhibitor cocktails (Roche, Basel, Switzerland). Protein concentration was determined using Pierce BCA Protein Assay Kit (Thermo Scientific, Reinach, Switzerland). The cell lysates were separated on 6 and 12% sodium dodecyl sulfate-polyacrylamide gels. Proteins were transferred to polyvinylidene fluoride membrane (Millipore, MA, USA). The membrane was blocked with 5% non-fat dry milk in Tris-buffered saline with Tween-20 (TBST) for 2 h at room temperature and then incubated for 8 h at 4 °C with the following antibodies: LC3 (1:1000, Abcam), p62 (1:1000, Proteintech), p53 (1:1000, Proteintech), AMPK (1:1000, Proteintech), p-AMPK (Thr172; 1:1000, Proteintech), p-mTOR (Ser2448; 1:1000, Cell Signaling, Danvers, MA, USA), mTOR (1:1000, Cell Signaling), p-p70S6K (Thr389; 1:1000, Cell Signaling), p70S6K (1:1000, Cell Signaling) and HDM2 (1:1000, Abcam). GADPH (1:1000, Cell Signaling) and Histone H3 (1:1000 Cell Signaling) antibodies were used as loading control. For detection, membranes were incubated with horseradishperoxidase-labeled goat anti rabbit secondary antibody (1:5000, Proteintech) and proteins were detected by ECL (Millipore). The relative quantity of the proteins was analyzed with the Image J software.

### Immunofluorescence staining

Cells on coverslips were fixed in 4% paraformaldehyde, washed in PBS containing 0.2% Triton X-100 and blocked in 10% normal goat serum. Cells then were stained with rabbit anti-LC3 (Abcam, 1: 200) antibodies overnight at 4 °C followed with Cy3-labeled secondary antibody (1:1000, Beyotime) for 1 h at room temperature. Nuclei were stained with DAPI. Fluorescence images were visualized with a BX53 Olympus fluorescence microscope. In this study, <5 puncta of LC3 was considered to reflect the basal level of autophagy in HUVECs. Cells containing >5 puncta of LC3 were considered to have increased level of autophagy.

### Statistical analysis

All data were presented as means ± s.d. of three independent experiments. Statistical comparisons of the results were done using Student’s *t-*test or one-way analysis of variance. A *p*-value < 0.05 is considered as statistically significant.
